# Supplementary material for: Intracellular Salmonella hijacks the mitochondrial citrate carrier to evade host oxidative defenses
Source: Nat Commun. 2025 Nov 6;16:9806. doi: 10.1038/s41467-025-64779-z (PMC12592408; doi:10.1038/s41467-025-64779-z)
Supplement: Supplementary file 1 — Supplementary information file [file 41467_2025_64779_MOESM1_ESM.pdf]

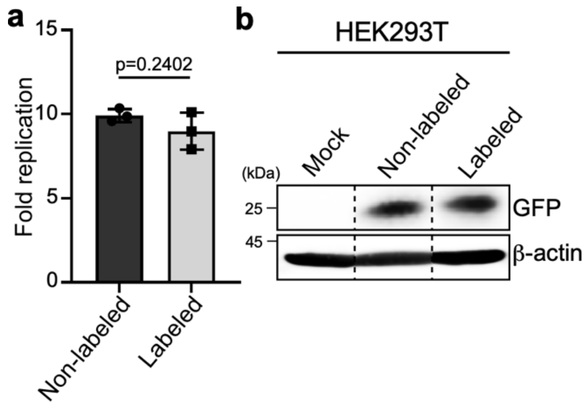

Supplementary Figure S1. **Intracellular growth and protein expression of magnetic bead-labeled *Salmonella*.** (**a**) Intracellular replication of bead-labeled versus unlabeled *Salmonella* Typhimurium (*S*. Typhimurium) in HEK293T cells infected at an MOI of 10. Colony-forming units (CFUs) were determined at 1 h and 24 h post-infection, and fold replication was calculated as the difference between CFUs at 1 and 24 hrs. Data are shown as mean ± s.d. from three independent experiments. Statistical analysis was performed using an unpaired two-sided *t*-test. (**b**) SCV maturation assessed by a SPI-2 reporter. HEK293T cells were infected for 16 h with bead-labeled or unlabeled bacteria carrying a *pagC* promoter–sfGFP fusion. Cell lysates were analysed by immunoblotting with anti-GFP and anti-β-actin (loading control). Dotted lines indicate where lanes from the same gel were spliced for presentation.

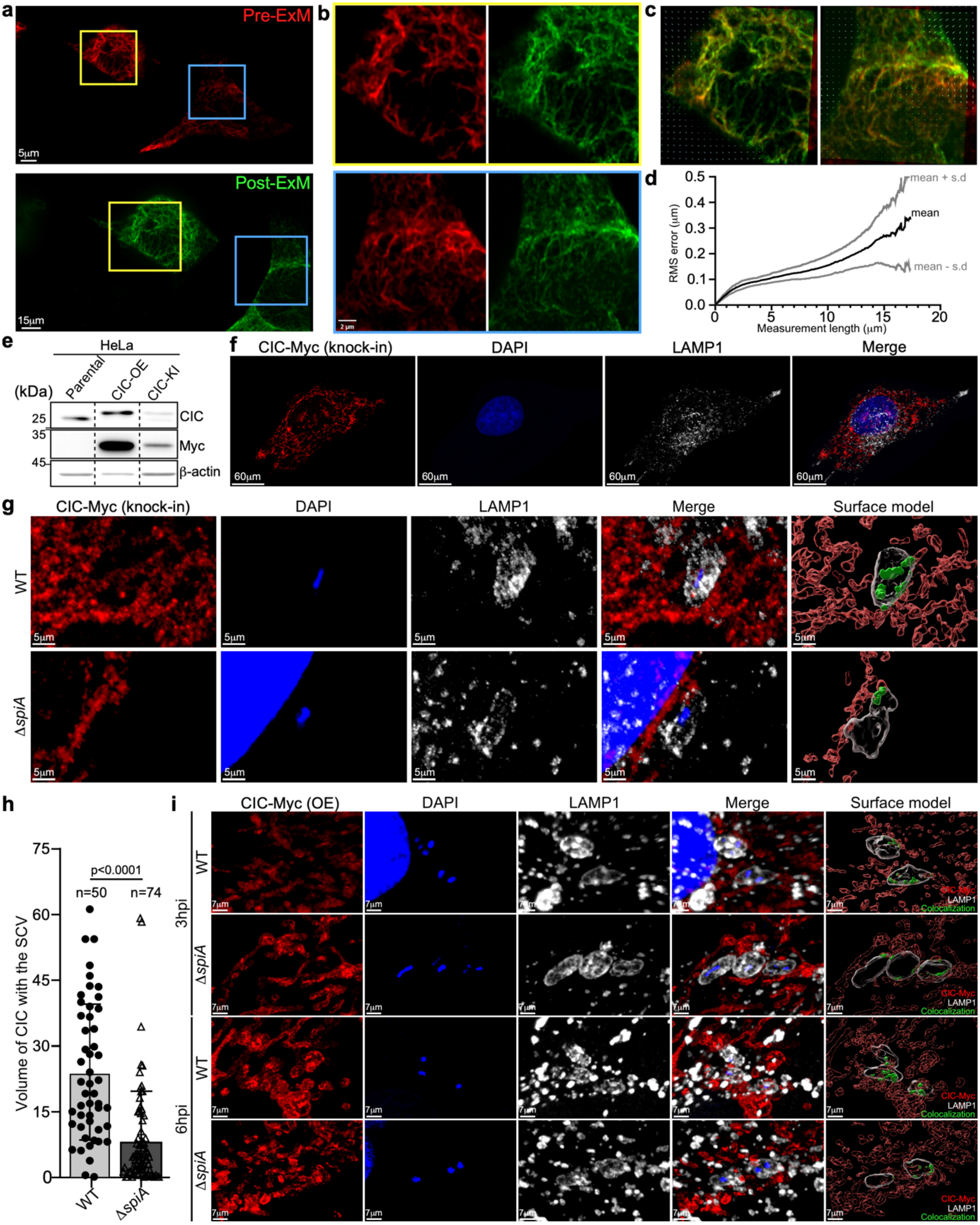

Supplementary Figure S2. **Expansion microscopy (ExM) shows that ClC colocalizes with the SCV membrane in a SPI-2–dependent manner.** (**a**-**d**) Evaluation of sample distortion after expansion microscopy. (**a**) Confocal images of vimentin immunostaining acquired before (Pre-Ex) and after (Post-Ex) expansion. (**b**) Enlarged views of boxed regions in (**a**). (**c**) Overlay of pre-expansion (red) and post-expansion (green) images after similarity and B-spline registration with Elastix. Arrow length and direction indicate the transformations required for optimal alignment. (**d**) Distortion analysis calculated as root-mean-square (RMS) length errors within 50 µm × 50 µm regions (mean, black; s.d., gray). Registration and RMS

calculations were performed with a customized Python script adapted from Chozinski et al. (PMID: 27064647). The code is available at https://github.com/AnChiLuo/Deformation-estimation-for-ExMicroscopy-Image. (**e**) Western blot analysis of Myc-tagged CIC in HeLa cell lines, with β-actin as a loading control. Lane designations: parental HeLa, HeLa stably overexpressing Myc-tagged CIC (CIC-OE), and HeLa with an endogenous Myc knock-in at the *SLC25A1* locus (CIC-KI). Dotted lines indicate where lanes from the same gel were spliced for presentation. (**f**) Expansion microscopy of CIC-KI HeLa cells. Mock-treated cells were stained with DAPI (blue), LAMP1 (white), and Myc (red), then imaged on a Leica TCS SP8 STED 3X confocal microscope. (**g**, **h**) Expansion microscopy of CIC-KI HeLa cells infected with *S.* Typhimurium. CIC-KI HeLa cells were infected for 24 h with either wild-type (WT) or Δ*spiA S.* Typhimurium and then stained with DAPI (blue), anti-LAMP1 (white), and anti-Myc (red) as in panel (**g**). *Salmonella*-containing vacuoles (SCVs) were identified by DAPI-positive bacteria within LAMP1-positive compartments. Images were processed in Imaris (Surface module) to generate three-dimensional reconstructions; green surfaces mark regions where CIC colocalizes with SCVs. Quantitative colocalization data are presented in panel (**h**). The WT image and analysis are shared with Supplementary Figure 10d and 10e, as all were derived from the same experiment performed in parallel. Data represent the mean ± SEM from three independent experiments; statistics were evaluated with an unpaired two-tailed *t*-test. Scale bar, 5 µm. (**i**) Representative images of Figure 1e. HeLa cells stably overexpressing Myc-tagged CIC (CIC-OE) were infected with either WT *S.* Typhimurium or the Δ*spiA* mutant. At 3 h and 6 h post-infection, cells were fixed, expanded according to the ExM protocol, and stained. Expanded samples were imaged on a Leica TCS SP8 STED 3X confocal microscope using identical acquisition settings for all conditions. Three-dimensional reconstructions were generated with the Surface module in Imaris. Green surface renderings indicate regions where CIC signal overlaps with LAMP1-positive, DAPI-positive vacuoles, denoting colocalization of CIC with SCVs. Scale bar, 7 µm.

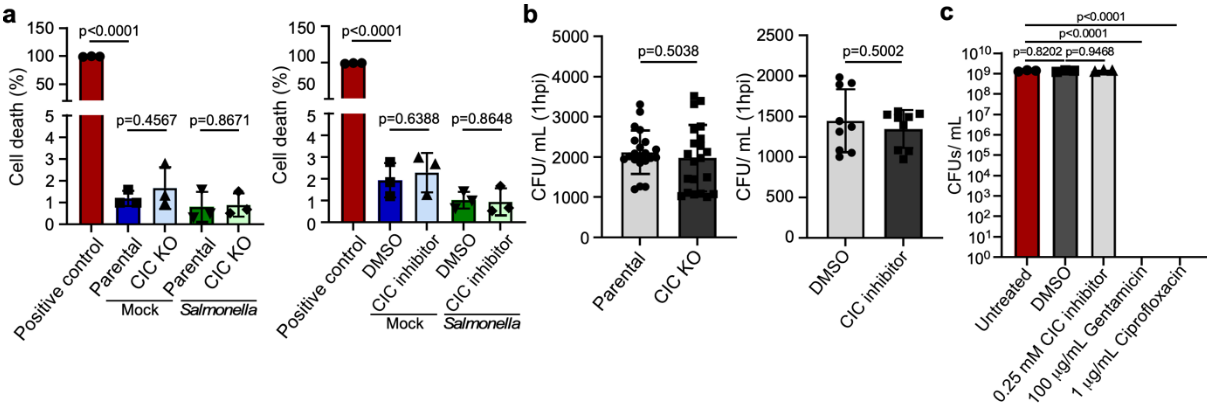

Supplementary Figure S3. **Impact of CIC disruption on host-cell physiology and intracellular *Salmonella* growth.** (**a**) Assessment of host cell viability after CIC disruption and *Salmonella* infection. RAW264.7 macrophages were either (left) CIC-deficient or parental, or (right) pretreated with DMSO or the CIC inhibitor. All cells were infected with *Salmonella* Typhimurium (*S.* Typhimurium) at an MOI of 5. At 24 h post-infection, viability was quantified by flow cytometry using Zombie Green; paraformaldehyde-fixed cells served as a positive control for dead cells. The percentage of Zombie-positive cells (dead) is shown as mean ± s.d. from three independent experiments, with at least 10,000 events analyzed per sample. Statistical analysis was performed using an unpaired two-sided *t*-test. (**b**) Assessment of *Salmonella* entry into RAW264.7 macrophages. Parental RAW264.7 cells, CIC-knockout (KO) cells, and parallel cultures pre-treated with either DMSO or a CIC inhibitor were infected with *S.* Typhimurium at an MOI of 5. Intracellular bacteria were recovered 1 h post-infection, and colony-forming units (CFUs) were enumerated to quantify bacterial entry. Data are shown as mean ± s.d from three independent experiments. Each circle and square represents a technical replicate from all experiments. Statistical analysis was performed using an unpaired two-sided *t*-test. (**c**) Effect of the CIC inhibitor on extracellular *Salmonella* growth. An overnight culture of *S.* Typhimurium was diluted 1:20 in cation-adjusted Mueller–Hinton broth (CAMHB) and grown to OD_{600}=0.9. The culture was then adjusted to 5 × 10^5 CFU mL^{-1} and incubated for 24 h with either the CIC inhibitor (0.25 mM) or DMSO. Gentamicin (100 µg mL^{-1}) and ciprofloxacin (1 µg mL^{-1}) served as positive growth-inhibition controls. CFUs were enumerated after incubation and are shown as mean ± s.d. from three independent experiments. Statistical analysis was performed using an unpaired two-sided *t*-test.

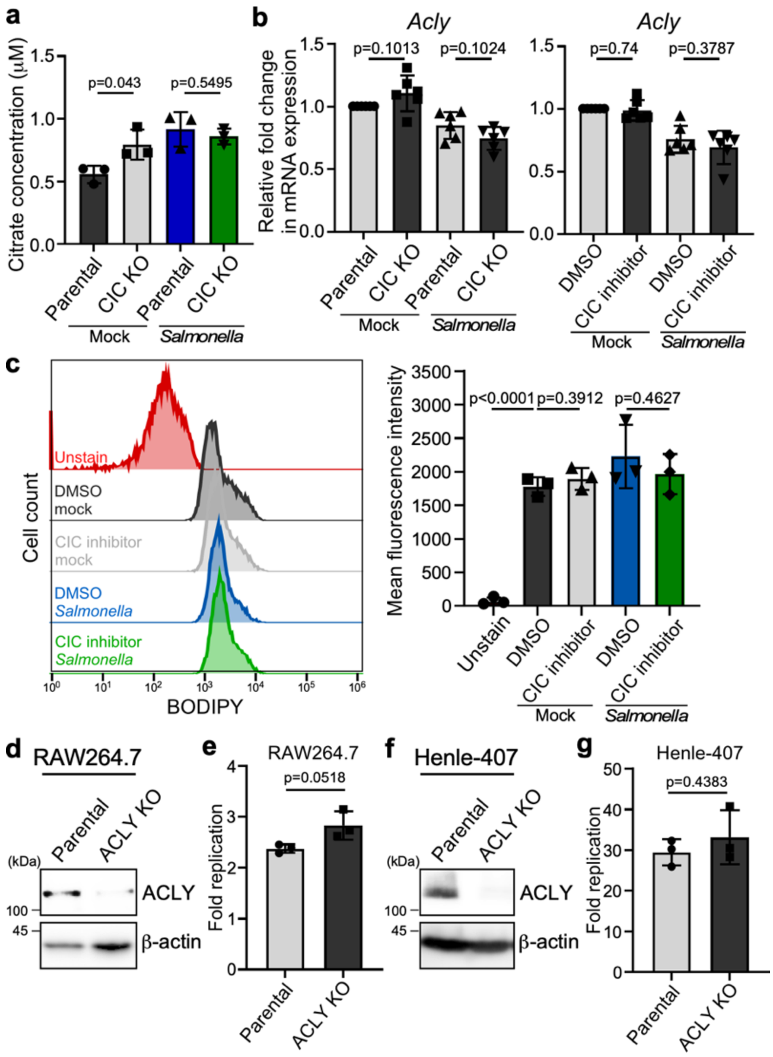

Supplementary Figure S4. Impact of CIC dysfunction on mitochondrial citrate levels and cellular lipid metabolism. (**a**) Measurement of mitochondrial citrate in CIC-deficient RAW264.7 cells. Parental and CIC-knockout (KO) RAW264.7 macrophages were infected with *S.* Typhimurium at a multiplicity of infection (MOI) of 5. At 24 h post-infection, mitochondria were isolated and citrate levels quantified. Data represent mean ± s.d. from three independent experiments. Statistical analysis was performed using an unpaired two-sided *t*-test. (**b**) RT-qPCR analysis of *Acly* expression in RAW264.7 macrophages. Parental and CIC-deficient RAW264.7 cells (left panel), as well as DMSO- or CIC inhibitor–treated cells (right panel), were infected with *S.* Typhimurium at an MOI of 5. Total RNA was extracted, and *Acly* transcript levels were quantified by RT-qPCR. Data are presented as the mean ± s.d. from six independent experiments. Statistical analysis was performed using an unpaired two-sided *t*-test. (**c**) Evaluation of lipid droplet accumulation in DMSO- and CIC inhibitor–treated RAW264.7 cells. RAW264.7 cells were infected with *S.* Typhimurium at an MOI of 5. Lipid droplet content in both mock-infected and infected cells was assessed 24 hours post-infection using flow cytometry with BODIPY staining. Mean fluorescence intensity (MFI) values are presented as the mean ± s.d. from three independent experiments. At least 10,000 cells were analyzed per condition. Statistical analysis was performed using an unpaired two-sided *t*-test. (**d-g**) Western blot analysis of ACLY expression in RAW264.7 (**d**) and Henle-407 (**f**) ACLY knockout (KO) cell lines, with β-actin used as a loading control. Cells were infected with *S.* Typhimurium at an MOI of 5, and intracellular bacterial growth was evaluated by calculating fold replication based on colony-forming units (CFUs) at 1 and 24 hours post-infection. (**e**, **g**) Data are presented as the mean ± s.d. from three independent experiments. Statistical analysis was performed using an unpaired two-sided *t*-test.

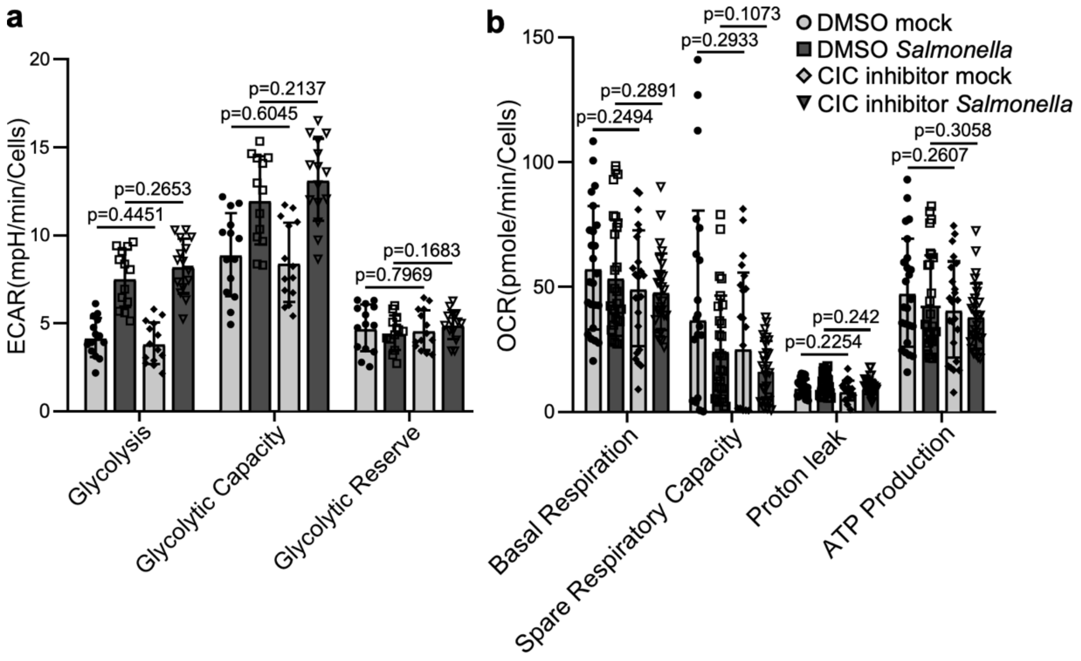

Supplementary Figure S5. **CIC inhibition had no significant effect on glycolytic activity or mitochondrial respiration.** Seahorse analysis was used to measure extracellular acidification rate (ECAR) parameters, including glycolysis, glycolytic capacity, and glycolytic reserve (**a**), as well as oxygen consumption rate (OCR) parameters, including basal respiration, spare respiratory capacity, proton leak, and ATP production (**b**). Parental RAW264.7 cells were pre-treated for 6 hours with either DMSO or the CIC inhibitor (0.25 mM), followed by infection with wild-type *Salmonella* or mock treatment. Cells were then subjected to analysis. Data are presented as the mean ± s.d. from three (**a**) and six (**b**) independent experiments. Statistical analysis was performed using an unpaired two-sided *t*-test.

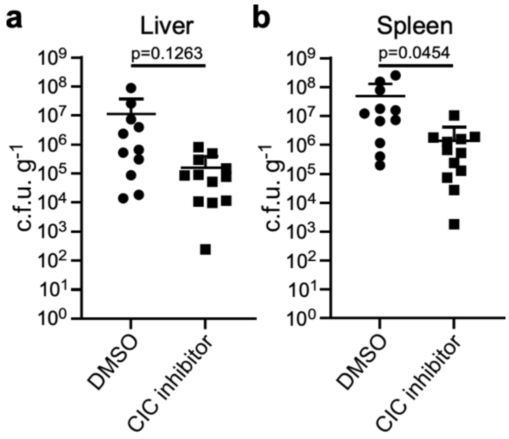

Supplementary Figure S6. **Therapeutically administration of the CIC inhibitor reduces the *Salmonella* burden in infected mice.** C57BL/6 mice were intraperitoneally infected with wild-type *Salmonella* Typhimurium (10^2 CFUs) and treated with the CIC inhibitor or DMSO every other day. Five days after infection, bacterial loads in the liver **(a)** and spleen **(b)** were determined. Each circle or square represents the CFU count of an individual animal. Data are presented as mean ± s.d. from three independent experiments. Statistical analysis was conducted using unpaired two-sided *t*-tests.

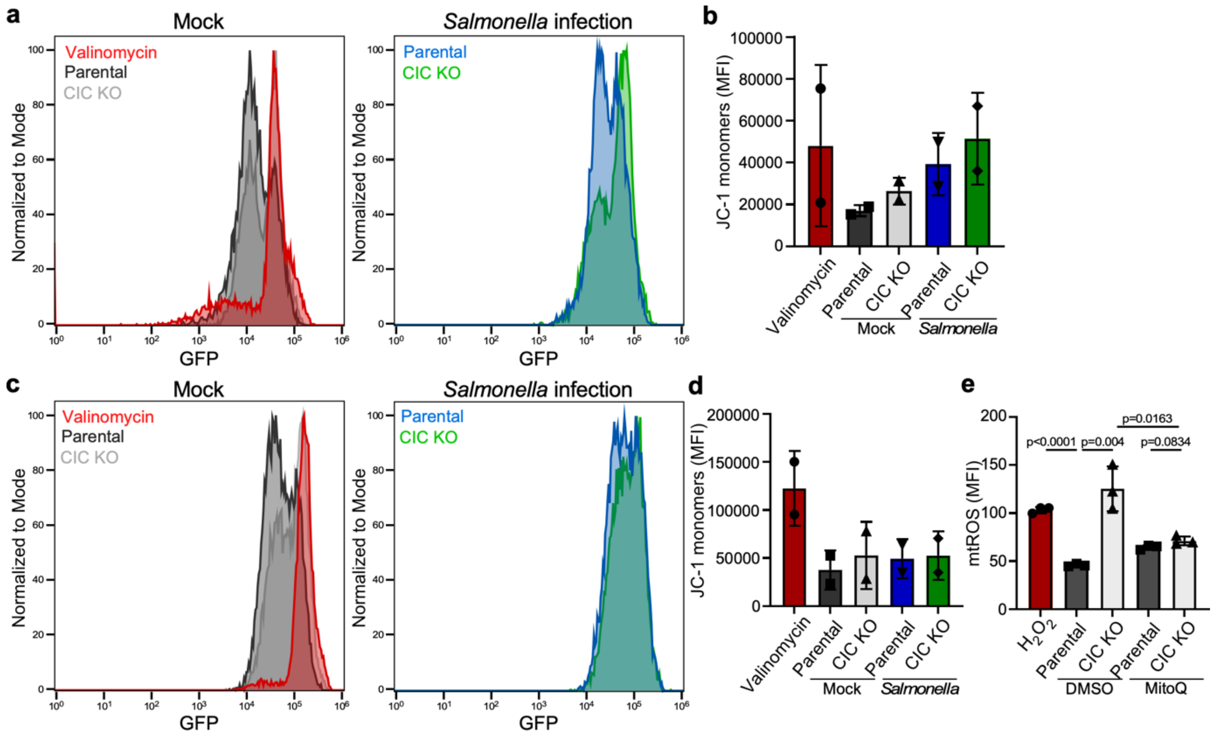

Supplementary Figure S7. **Assessment of mitochondrial membrane potential and mitochondrial ROS levels during infection.** (**a-d**) Mitochondrial membrane potential was assessed in CIC-knockout (KO) RAW264.7 cells (**a, b**) and Henle-407 cells (**c, d**) alongside their respective parental lines. Cells were infected with *S.* Typhimurium at a multiplicity of infection (MOI) of 20. Sixteen hours post-infection, cells were stained with JC-1 dye and analyzed by flow cytometry. Mean fluorescence intensity (MFI) of JC-1 monomers reflects mitochondrial depolarization. At least 10,000 cells were analyzed per condition. Data are showned from two independent experiments. (**e**) Mitochondrial ROS (mtROS) levels were evaluated in infected parental and CIC-KO RAW264.7 cells treated with the mitochondrial-targeted antioxidant MitoQ (0.5 μM). Cells were infected with *S.* Typhimurium expressing sfGFP (MOI = 20), and mtROS levels in mock and GFP-positive infected cells were measured 24 hours post-infection using MitoSOX Red and flow cytometry. Data represent mean ± s.d. from three independent experiments. Statistical analysis was performed using an unpaired two-sided *t*-test.

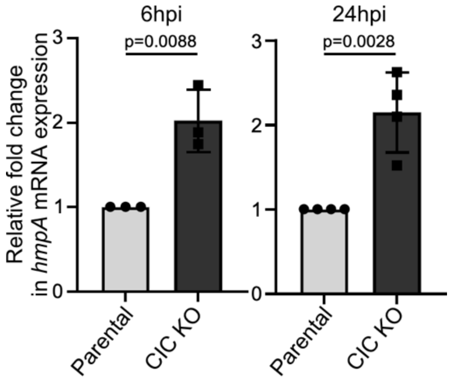

Supplementary Figure S8. RT-qPCR analysis of the mRNA level in *Salmonella* oxidative stress response genes within host cells. Parental and CIC-knockout (KO) RAW264.7 macrophages were infected with *Salmonella* Typhimurium at a multiplicity of infection (MOI) of 100. Six and twenty-four hours after infection, mRNA was isolated and analyzed by RT-qPCR using primers targeting *hmpA*. Data are presented as the mean ± s.d. from three (6 hpi) and four (24 hpi) independent experiments. Statistical analysis was performed using an unpaired two-sided *t*-test.

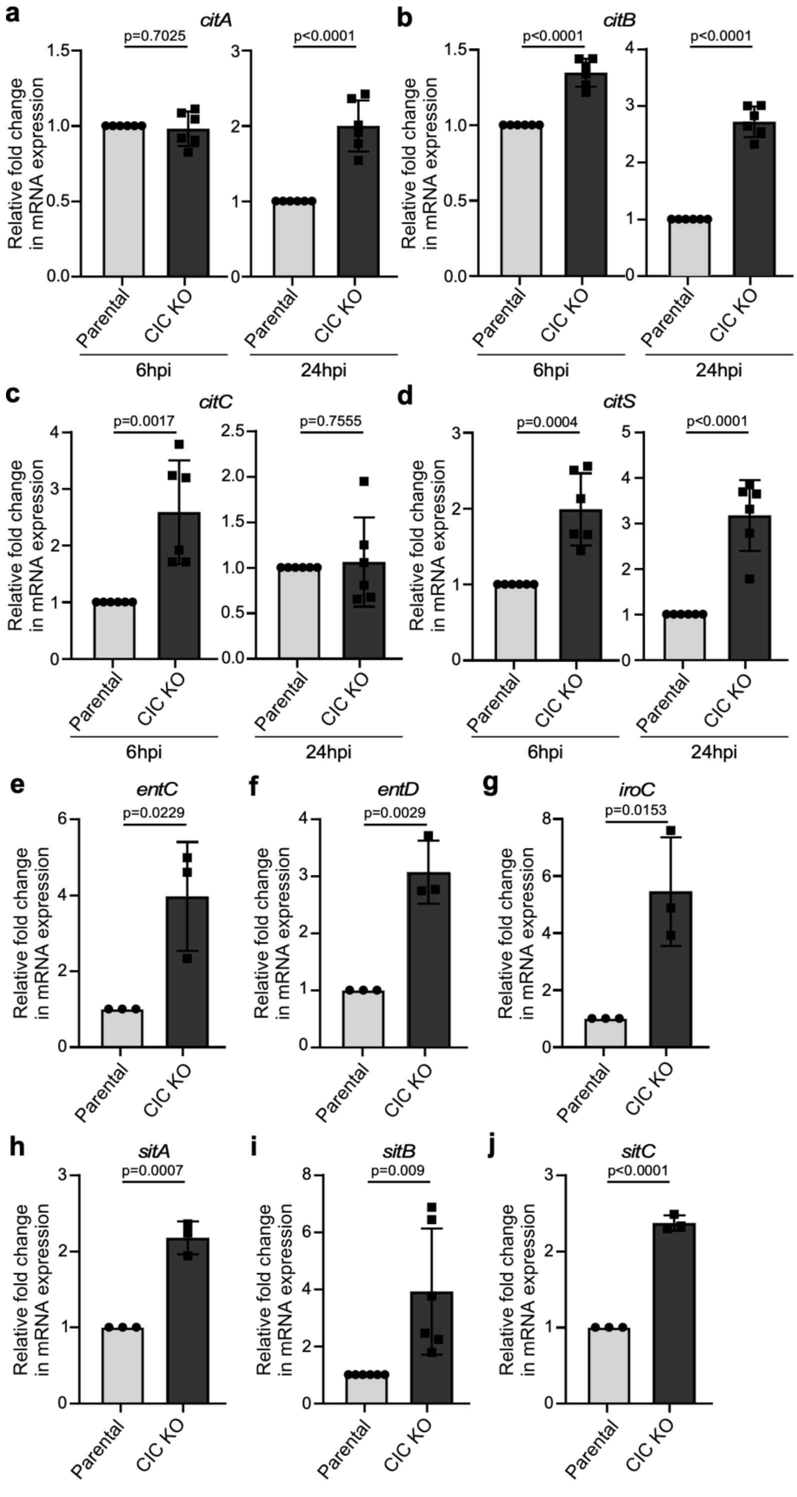

Supplementary Figure S9. RT-qPCR analysis of mRNA levels of *Salmonella* genes responsive to citrate and iron. Parental and CIC-knockout (KO) RAW264.7 macrophages were infected with *Salmonella* Typhimurium at a multiplicity of infection (MOI) of 100. Total RNA was isolated at 6 and 24 hours post-infection and analyzed by RT-qPCR. Transcript levels of *citA* (**a**), *citB* (**b**), *citC* (**c**), and *citS* (**d**) were measured at both time points. Expression of iron-regulated genes *entC* (**e**), *entD* (**f**), *iroC* (**g**), *sitA* (**h**), *sitB* (**i**), and *sitC* (**j**) was assessed at 24 hours post-infection. Each circle or square represents an individual replicate. Data are presented as mean ± s.d. Panels **a-d** represent data from six independent experiments; panels **e**, **f**, **g**, **h**, and **j** from three independent experiments; and panel **i** from six independent experiments. Statistical analysis was performed using an unpaired two-sided *t*-test.

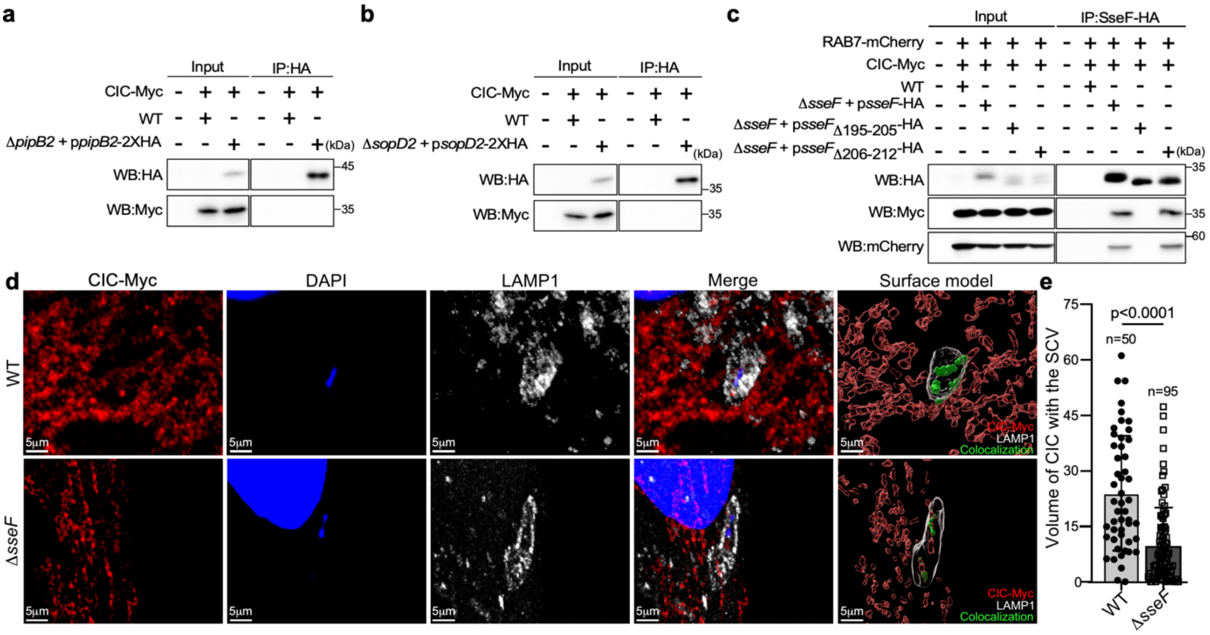

Supplementary Figure S10. Analysis of the impact of SPI-2 effectors on CIC recruitment to the *Salmonella*-containing vacuole (SCV). (**a, b**) HEK293T cells transiently transfected with a plasmid-expressing Myc-tagged CIC were infected with either wild-type (WT) *S.* Typhimurium, *S.* Typhimurium expressing HA-epitope tagged PipB2, or HA-epitope tagged SopD2 at a multiplicity of infection (MOI) of 20. At twenty-four hours post-infection, cell lysates were subjected to immunoprecipitation using anti-HA Sepharose beads. The immunoprecipitates were examined by Western blot analysis. IP, immunoprecipitate. (**c**) HEK293T cells transiently co-transfected with plasmids encoding Myc-tagged CIC and RAB7–mCherry were infected with WT S. Typhimurium, or with ΔsseF mutant strains expressing HA-tagged full-length SseF, SseF lacking amino acids 195–205, or SseF lacking amino acids 206–212. Sixteen hours post-infection, cell lysates were subjected to immunoprecipitation followed by Western blotting. (**d**, **e**) CIC-knockin (KI) HeLa cells were infected for 24 hours with either WT *S.* Typhimurium or the Δ*sseF* mutant strain (both shown in blue). The WT data shown here are identical to those in Supplementary Figure 2g-h, as all three conditions (WT, Δ*spiA*, Δ*sseF*) were processed and analyzed in the same experiment. Following infection, cells were stained with DAPI (blue), anti-LAMP1 (white), and anti-Myc (red), then imaged and analyzed to assess CIC colocalization with SCVs. SCVs were identified as DAPI-positive bacteria enclosed within LAMP1-positive compartments. Three-dimensional surface models were generated using the Surface module in Imaris, where green regions indicate colocalization between CIC and SCVs, and white regions mark LAMP1-positive vacuoles. Quantification of colocalization at 24 hours post-infection is shown in (e). Data represent the mean ± SEM from three independent experiments. Statistical significance was determined by unpaired two-sided *t*-tests. Scale bar: 5 μm.

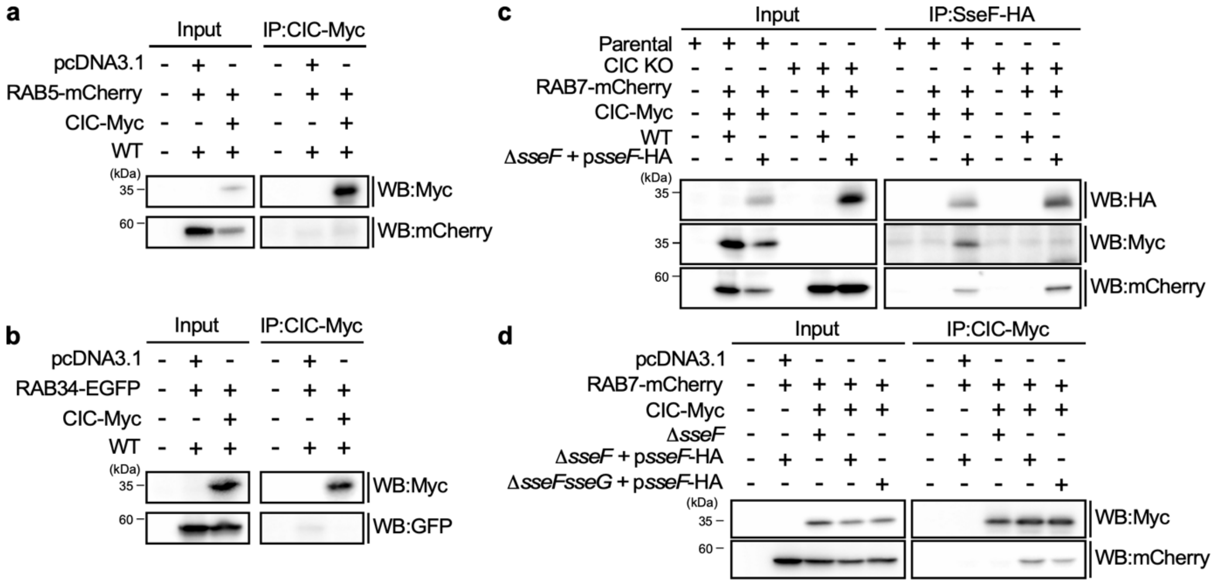

Supplementary Figure S11. Analysis of the specificity of CIC interactions with RAB GTPases and SPI-2 effectors. (**a**, **b**) HEK293T cells were transiently transfected with plasmids expressing Myc-tagged CIC and mCherry-RAB5 (**a**) or EGFP-RAB34 (**b**). Following infection with wild-type (WT) *S.* Typhimurium at a multiplicity of infection of 20, cells were lysed at 24 hours post-infection and subjected to immunoprecipitation using anti-Myc Sepharose beads. The resulting immunoprecipitates were analyzed by immunoblotting with anti-Myc, anti-mCherry, and anti-GFP antibodies. (**c**) Parental and CIC-knockout (KO) HEK293T cells, transiently co-transfected with plasmids encoding Myc-tagged CIC and mCherry-RAB7, were infected with either WT *S.* Typhimurium or the Δ*sseF* mutant expressing HA-tagged SseF at a multiplicity of infection of 20. At 24 hours post-infection, cell lysates were subjected to immunoprecipitation using anti-HA Sepharose beads, followed by western blotting with antibodies against HA, mCherry, and Myc. (**d**) HEK293T cells were transiently transfected with plasmids encoding Myc-tagged CIC and mCherry-RAB7, then infected with either the Δ*sseF Salmonella* strain, a Δ*sseF* strain expressing HA-tagged SseF, or a Δ*sseF*Δ*sseG* strain expressing HA-tagged SseF. At 24 hours post-infection, cells were lysed and subjected to immunoprecipitation using anti-Myc Sepharose beads, followed by immunoblotting with antibodies against Myc and mCherry. IP, immunoprecipitates.

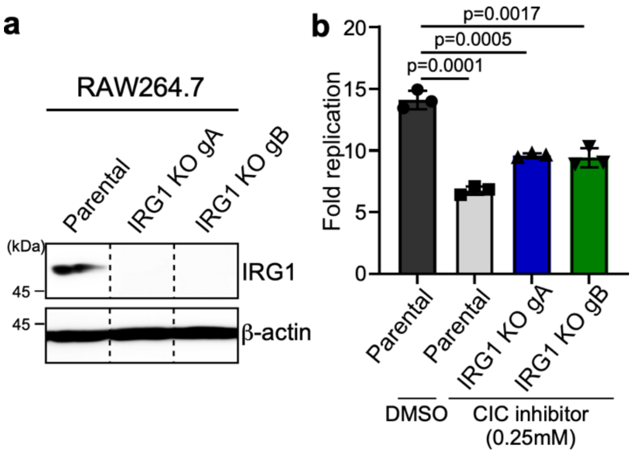

Supplementary Figure S12. Itaconate is dispensable for controlling intracellular *Salmonella* replication in cells with impaired CIC function. (**a**) Western blot analysis of IRG1-knockout (KO) cell lines generated via CRISPR/Cas9 genome editing. Two independent clones were produced using distinct guide RNAs (gA and gB) targeting the IRG1 gene. β-actin was used as a loading control. Dotted lines indicate where lanes from the same gel were spliced for presentation. (**b**) Cells were pretreated with either DMSO or CIC inhibitor and infected with *Salmonella* Typhimurium at a multiplicity of infection (MOI) of 5. Colony-forming units (CFUs) were quantified at 1 and 24 hours post-infection. Fold replication was determined by calculating the difference in CFUs between 1 and 24 hours. Data are presented as mean ± s.d from three independent experiments. Statistical analysis was performed using an unpaired two-sided *t*-test.

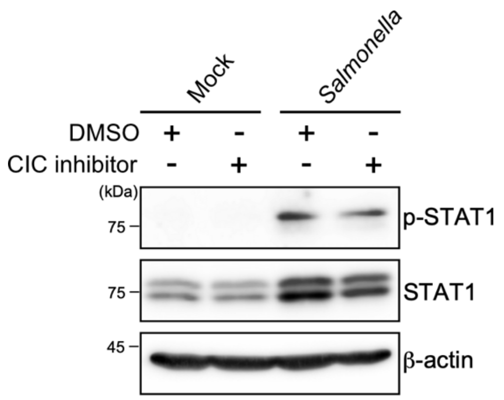

Supplementary Figure S13. Activation of STAT1 signaling in cells with impaired CIC function during *Salmonella* infection. RAW264.7 cells pre-treated with DMSO or the CIC inhibitor (0.25 mM), were infected with *S.* Typhimurium at a multiplicity of infection (MOI) of 5 for 24 hours. Cell lysates were collected and analyzed by western blotting using antibodies against phosphorylated STAT1 (p-STAT1), total STAT1, and β-actin (loading control).

Supplementary Table 1. All *Salmonella* strains used in this study

Strain	Description	Source
CGU03	*Salmonella* Typhimurium SL1344 strain	Chang Gung University
NTUMT24	*Salmonella* Typhimurium 14028 strain	ATCC
SJ LAB	SL1344, pWSK-sfGFP	This study
SJ LAB	SL1344, pWSK-mCherry	This study
pRA69	SL1344, pBAD24-Nluc-*soxS*	This study
pRA70	SL1344, pBAD24-*oxyR*-Nluc	This study
pRA83	SL1344, pBAD24-*katG*-Nluc	This study
RA22	SL1344 ∆*sseFsseG*, pWSK-*sseF*-HA	This study
RA26	SL1344, pBAD24-p*iroB*-Nluc	This study
RA27	SL1344 ∆*sseF*	This study
RA28	SL1344 ∆*sseF*, pWSK-*sseF*-HA	This study
RA3	SL1344 ∆*spiA*	This study
RA32	SL1344 ∆*sseF*, pWSK-*sseF* ∆*195-205*-HA	This study
RA33	SL1344 ∆*sseF*, pWSK-*sseF* ∆*206-212*-HA	This study
RA34	SL1344, pFCcGi	This study
RA37	SL1344-p*pagC*-sfGFP	This study
RA73	SL1344 ∆*sopD2*, pWSK- *sopD2*-2X HA	This study
RA74	SL1344 ∆*pipB2*, pWSK- *pipB2*-2X HA	This study
RA75	SL1344, pBAD24-p*citS*-Nluc	This study
RA76	SL1344, pBAD24-pton*B*-Nluc	This study
RA77	SL1344, pBAD24-p*stm1485*-Nluc	This study

Supplementary Table 2. All the plasmids used in this study

Plasmid	Description	Source
pBJ104	pAcGFP1-mCherry-Rab5	Dr. Wen Chang / Academia Sinica
pBJ105	pAcGFP1-EGFP-Rab34	Dr. Wen Chang / Academia Sinica
pBJ13	pcDNA3.1	Dr. Wen Chang / Academia Sinica
pBJ2	pAcGFP1-Rab7-mCherry	Dr. Wen Chang / Academia Sinica
pBJ23	pWSK-sfGFP	Dr. Jorge E. Galán / Yale University
pBJ24	pWSK-mcherry	Dr. Jorge E. Galán / Yale University
pBJ78	pFCcGi	Addgene #59324
pRA112	pSIN-CTP-Myc	This study
pRA61	pcDNA3.1-CIC-Myc	This study

Flow cytometry gating strategy

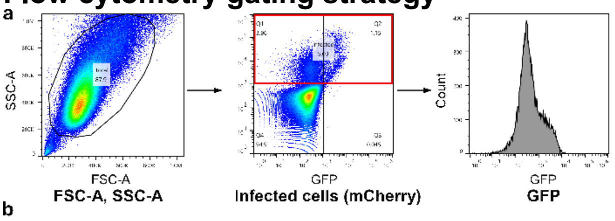

(a) Used in Figure 2c (pFCcGi)

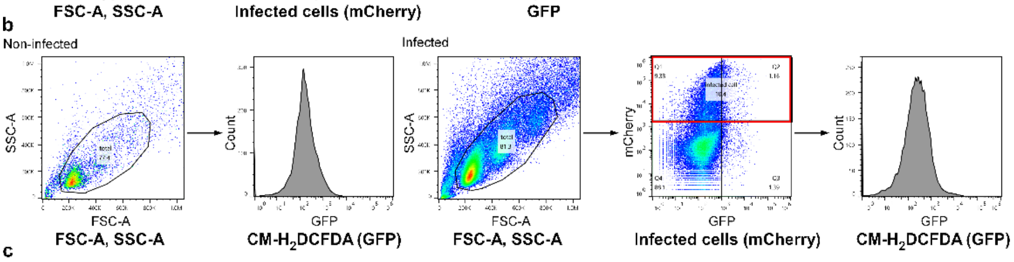

(b) Used in Figure 3d (CM-H_2DCFDA).

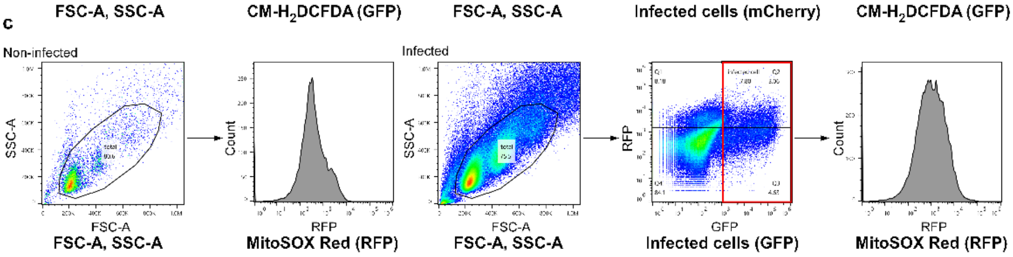

(c) Used in Figure 3a (MitoSOX Red).

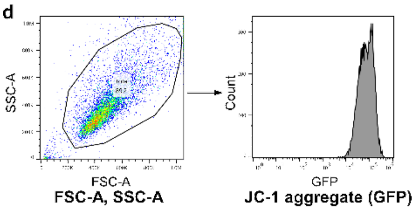

(d) Used in Supplementary Figure S7 (JC-1).

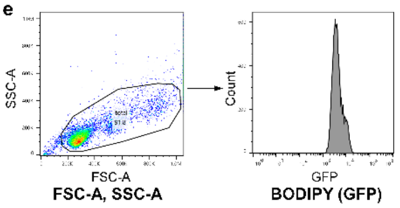

(e) Used in Supplementary Figure S4c (BODIPY).

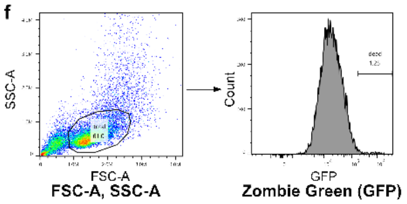

(f) Used in Supplementary Figure S3a (Zombie Green).
