# Supplementary material for: Intracellular Salmonella hijacks the mitochondrial citrate carrier to evade host oxidative defenses
Source: Nat Commun. 2025 Nov 6;16:9806. doi: 10.1038/s41467-025-64779-z (PMC12592408; doi:10.1038/s41467-025-64779-z)
Supplement: Supplementary file 2 — Description of Additional Supplementary File [file 41467_2025_64779_MOESM2_ESM.pdf]

Description of Additional Supplementary Files

Supplementary Movie 1: Three-dimensional reconstruction of HeLa cells stably expressing Myctagged CIC (red) and stained with TOM20 (white) and DAPI (blue). Cells were infected with Salmonella Typhimurium constitutively expressing sfGFP (green). The movie shows CIC localization and its spatial association with Salmonella and mitochondria under infection conditions, corresponding to panel c of Figure 1

Supplementary Data 1: All antibodies, chemical reagents, and oligonucleotide sequences used in this study are listed in the file.
