# Supplementary material for: Intracellular Salmonella hijacks the mitochondrial citrate carrier to evade host oxidative defenses
Source: Nat Commun. 2025 Nov 6;16:9806. doi: 10.1038/s41467-025-64779-z (PMC12592408; doi:10.1038/s41467-025-64779-z)
Supplement: Supplementary file 5 — Reporting summary [file 41467_2025_64779_MOESM5_ESM.pdf]

nature portfolio

Corresponding author(s): Shu-Jung Chang

Last updated by author(s): Sep 19, 2025

Reporting Summary

Nature Portfolio wishes to improve the reproducibility of the work that we publish. This form provides structure for consistency and transparency in reporting. For further information on Nature Portfolio policies, see our Editorial Policies and the Editorial Policy Checklist.

Statistics

For all statistical analyses, confirm that the following items are present in the figure legend, table legend, main text, or Methods section.

n/a	Confirmed	
☐	☒	The exact sample size (*n*) for each experimental group/condition, given as a discrete number and unit of measurement
☐	☒	A statement on whether measurements were taken from distinct samples or whether the same sample was measured repeatedly
☐	☒	The statistical test(s) used AND whether they are one- or two-sided<br>*Only common tests should be described solely by name; describe more complex techniques in the Methods section.*
☒	☐	A description of all covariates tested
☐	☒	A description of any assumptions or corrections, such as tests of normality and adjustment for multiple comparisons
☐	☒	A full description of the statistical parameters including central tendency (e.g. means) or other basic estimates (e.g. regression coefficient) AND variation (e.g. standard deviation) or associated estimates of uncertainty (e.g. confidence intervals)
☐	☒	For null hypothesis testing, the test statistic (e.g. *F*, *t*, *r*) with confidence intervals, effect sizes, degrees of freedom and *P* value noted<br>*Give P values as exact values whenever suitable.*
☒	☐	For Bayesian analysis, information on the choice of priors and Markov chain Monte Carlo settings
☒	☐	For hierarchical and complex designs, identification of the appropriate level for tests and full reporting of outcomes
☒	☐	Estimates of effect sizes (e.g. Cohen's *d*, Pearson's *r*), indicating how they were calculated

Our web collection on statistics for biologists contains articles on many of the points above.

Software and code

Policy information about availability of computer code

Data collection	CFX Manager™ Software_Version 4.1.2433.1219 (gene expression), ChemiDoc MP Imaging System_Version 2.4.0.03 and Image Lab_Version 6.0.1 (imaging and quantification of the band intensity of western blot), Canvas X Draw_Version 20 & Adobe Photoshop_Version 26.6.0 (image preparation), FlowJo_Version 10.9.0 (analysis of flow cytometry data), ImageJ_Version 1.54f and Imaris_Version 9.9.1 (image analysis), Agilent Seahorse Wave software_Version 2.6.3.5, Agilent BioTek Gen5_Version3.16 (for measurement of citrate concentration), SoftMax Pro7_Version 7.1.0 (for Nanoluciferase), and BioRender. The codes used in this study are available on GitHub and archived on Zenodo (DOIs: https://doi.org/10.5281/zenodo.13373734; https://doi.org/10.5281/zenodo.13756849).
Data analysis	Statistic calculations were performed using GraphPad Prism V9. Images were processed and rendered by Imaris (Oxford).

For manuscripts utilizing custom algorithms or software that are central to the research but not yet described in published literature, software must be made available to editors and reviewers. We strongly encourage code deposition in a community repository (e.g. GitHub). See the Nature Portfolio guidelines for submitting code & software for further information.

Data

Policy information about availability of data

All manuscripts must include a data availability statement. This statement should provide the following information, where applicable:

- Accession codes, unique identifiers, or web links for publicly available datasets
- A description of any restrictions on data availability
- For clinical datasets or third party data, please ensure that the statement adheres to our policy

Source data are provided with this paper.

Research involving human participants, their data, or biological material

Policy information about studies with human participants or human data. See also policy information about sex, gender (identity/presentation), and sexual orientation and race, ethnicity and racism.

Reporting on sex and gender	N/A
Reporting on race, ethnicity, or other socially relevant groupings	N/A
Population characteristics	N/A
Recruitment	N/A
Ethics oversight	N/A

Note that full information on the approval of the study protocol must also be provided in the manuscript.

Field-specific reporting

Please select the one below that is the best fit for your research. If you are not sure, read the appropriate sections before making your selection.

☒ Life sciences ☐ Behavioural & social sciences ☐ Ecological, evolutionary & environmental sciences

For a reference copy of the document with all sections, see nature.com/documents/nr-reporting-summary-flat.pdf

Life sciences study design

All studies must disclose on these points even when the disclosure is negative.

Sample size	We have updated the Statistics & Reproducibility section to describe how sample sizes were chosen for all experiments. No statistical methods were used to predetermine sample size; rather, sample sizes were based on established practices in the field and are sufficient to ensure reproducibility, as detailed in the revised text. All experiments were biologically independently repeated for at least three times.
Data exclusions	No data was excluded.
Replication	At least three independent experiments were performed with similar results. The number of biological replicates were indicated in the figure legends.
Randomization	In all cases, samples (animals, cultured cell lines, etc) were randomly assigned to the different experimental groups.
Blinding	Investigators were not blinded, but this was not necessary as data were collected and examined by different investigators.

Reporting for specific materials, systems and methods

We require information from authors about some types of materials, experimental systems and methods used in many studies. Here, indicate whether each material, system or method listed is relevant to your study. If you are not sure if a list item applies to your research, read the appropriate section before selecting a response.

Materials & experimental systems

n/a	Involved in the study	
☐	☒	Antibodies
☐	☒	Eukaryotic cell lines
☒	☐	Palaeontology and archaeology
☐	☒	Animals and other organisms
☒	☐	Clinical data
☒	☐	Dual use research of concern
☒	☐	Plants

Methods

n/a	Involved in the study	
☒	☐	ChIP-seq
☐	☒	Flow cytometry
☒	☐	MRI-based neuroimaging

Antibodies

Antibodies used

Streptavidin, Alexa Fluor 568 conjugate; Thermo Fisher; Cat# S11226 (1:200)
Rabbit polyclonal anti-Stat1; Cell Signaling; Cat# 9172 (1:1000)
Rabbit monoclonal anti-phospho-Stat1; Cell Signaling; Cat# 9167 (1:1000)
Rabbit monoclonal anti-IRG1; ABcam; Cat# ab222411 (1:1000)
Mouse monoclonal Tom20 Antibody (F-10); Santa Cruz Biotechnology; Cat# sc-17764 (1:500)
Rabbit polyclonal anti-HA; GeneTex Cat# GTX115044 (1:1000)
Mouse monoclonal anti-HA; ABclonal Cat# AE008 (1:1000)
Rabbit monoclonal anti-Myc; ABclonal Cat# AE070 (1:2000 for western blot; 1:1000 for expansion microscope)
Mouse monoclonal anti-LAMP1; Cell Signaling Cat# 15665 (1:100)
Rabbit anti-GFP; GeneTex Cat# GTX113617 (1:1000)
Rabbit polyclonal anti-mCherry; GeneTex Cat# GTX128508 (1:10000)
Rabbit polyclonal anti-β-actin; ABclonal Cat# AC026 (1:10000)
Rabbit polyclonal anti-CIC; Proteintech Cat# 15235-1-AP (1:500)
HRP-linked goat anti-rabbit IgG; Cell Signaling Cat# 7074 (1:2000)
HRP-linked goat anti-mouse IgG; Cell Signaling Cat# 7076 (1:2000)
Peroxidase AffiniPure Goat Anti-Rabbit IgG; Jackson ImmunoResearch Cat# 115035003 (1:20000)
Goat anti-rabbit IgG alexa fluor 594; Thermo Fisher Cat# A11012 (1:2000)
Goat anti-mouse IgG alexa fluor 647; Thermo Fisher Cat# A21236 (1:2000)
ATP-Citrate Lyase Antibody; Cell Signaling Cat# 4332 (1:1000)
Streptavidin, Alexa Fluor 488 conjugate; Thermo Fisher Cat# S11223 (1:200)
Goat anti-mouse IgG (H+L) cross-absorbed secondary antibody, Biotin-XX; Thermo Fisher Cat# B2763 (1:200)
Goat anti-rabbit IgG (H+L) cross-absorbed secondary antibody, Biotin-XX; Thermo Fisher Cat# B2770 (1:200)
Abberior STAR 580, goat anti-rabbit IgG 500μl(1mg/ml); Abberior Cat# ST580-1002 (1:200)

Validation

All antibodies were purchased from reputable vendors and validated with positive or negative control in this study. Commercial antibodies were also validated by the manufacturers as stated in the manual instructions. Companies provide quality certificates and information about validation strategies.

Eukaryotic cell lines

Policy information about cell lines and Sex and Gender in Research

Cell line source(s)

Henle-407 originally obtained from the Roy Curtiss laboratory collection, were provided as a gift from Dr. Jorge Galan's lab. HEK293T(ATCC), RAW 264.7 (ATCC TIB-71), and HeLa cell (Bioresource Collection and Research Center in Taiwan)

Authentication

Cell lines were not authenticated.

Mycoplasma contamination

The cell lines were routinely tested with mycoplasma detection kits. All cell lines tested negative for mycoplasma contamination.

Commonly misidentified lines
(See ICLAC register)

N/A

Animals and other research organisms

Policy information about studies involving animals; ARRIVE guidelines recommended for reporting animal research, and Sex and Gender in Research

Laboratory animals

Male wild-type C57BL/6J mice, aged 6-8 weeks, were used in the experiments. All C57BL/6J mice were provided by National Taiwan University College of Medicine Laboratory Animal Center.

Wild animals

N/A

Reporting on sex

Described in the section of Methods

Field-collected samples	N/A
Ethics oversight	All animal experiments were conducted in strict accordance with the protocols approved by the policies of the National Taiwan University College of Medicine National Taiwan University (protocol number 20200228). The ethical committee that approved this study was led by Huei-Wen Chen (Graduate Institute of Toxicology, College of Medicine, National Taiwan University).

Note that full information on the approval of the study protocol must also be provided in the manuscript.

Plants

Seed stocks	N/A
Novel plant genotypes	N/A
Authentication	N/A

Flow Cytometry

Plots

Confirm that:

☒ The axis labels state the marker and fluorochrome used (e.g. CD4-FITC).

☒ The axis scales are clearly visible. Include numbers along axes only for bottom left plot of group (a 'group' is an analysis of identical markers).

☒ All plots are contour plots with outliers or pseudocolor plots.

☒ A numerical value for number of cells or percentage (with statistics) is provided.

Methodology

Sample preparation	All protocols of the FACS related experiments are described in detail in the "Methods" section. Typically, the FACS experiments involved staining with one to two colors, unless explicitly specified otherwise.
Instrument	Flow data was obtained by Attune NxT Flow Cytometers (Thermo Fisher)
Software	Data were analyzed with Flowjo V10.8.1.
Cell population abundance	RAW264.7 and Henle-407 cells
Gating strategy	RAW264.7 and Henle-407 populations were identified with FSC-A/SSC-A.

☒ Tick this box to confirm that a figure exemplifying the gating strategy is provided in the Supplementary Information.
